# Computational analysis of the functional and structural impact of the most deleterious missense mutations in the human Protein C

**DOI:** 10.1371/journal.pone.0294417

**Published:** 2023-11-28

**Authors:** Mahvash Farajzadeh-Dehkordi, Ladan Mafakher, Abbas Harifi, Fatemeh Samiee-Rad, Babak Rahmani

**Affiliations:** 1 Cellular and Molecular Research Center, Institute for Prevention of Non-Communicable Diseases, Qazvin University of Medical Sciences, Qazvin, Iran; 2 Department of Molecular Medicine, Qazvin University of Medical Sciences, Qazvin, Iran; 3 Thalassemia & Hemoglobinopathy Research Center, Health Research Institute, Ahvaz Jundishapur University of Medical Sciences, Ahvaz, Iran; 4 Department of Electrical and Computer Engineering, University of Hormozgan, Bandar Abbas, Iran; 5 Department of Pathobiology, Faculty of Medical School, Qazvin University of Medical Sciences, Qazvin, Iran; Cukurova University: Cukurova Universitesi, TURKEY

## Abstract

Protein C (PC) is a vitamin K-dependent factor that plays a crucial role in controlling anticoagulant processes and acts as a cytoprotective agent to promote cell survival. Several mutations in human PC are associated with decreased protein production or altered protein structure, resulting in PC deficiency. In this study, we conducted a comprehensive analysis of nonsynonymous single nucleotide polymorphisms in human PC to prioritize and confirm the most high-risk mutations predicted to cause disease. Of the 340 missense mutations obtained from the NCBI database, only 26 were classified as high-risk mutations using various bioinformatic tools. Among these, we identified that 12 mutations reduced the stability of protein, and thereby had the greatest potential to disturb protein structure and function. Molecular dynamics simulations revealed moderate alterations in the structural stability, flexibility, and secondary structural organization of the serine protease domain of human PC for five missense mutations (L305R, W342C, G403R, V420E, and W444C) when compared to the native structure that could maybe influence its interaction with other molecules. Protein-protein interaction analyses demonstrated that the occurrence of these five mutations can affect the regular interaction between PC and activated factor V. Therefore, our findings assume that these mutants can be used in the identification and development of therapeutics for diseases associated with PC dysfunction, although assessment the effect of these mutations need to be proofed in in-vitro.

## Introduction

Protein C (PC), a vitamin K-dependent plasma serine protease zymogen, is activated on endothelial cell surfaces by the thrombin-thrombomodulin (TM) complex. Activated protein C (APC) performs an essential function in regulating the coagulation cascade and thrombosis by cleaving and inhibiting procoagulant factors VIII and V with the assistance of protein S and various lipid surfaces. In addition to its anticoagulant mechanism, APC also exhibits cell signaling functions that contribute to multiple cytoprotective activities, including profibrinolytic, anti-apoptotic, and anti-inflammatory properties; epithelial and endothelial barrier protection; and pro-cell-survival effects. These functions are mediated by cell-specific and context-specific receptor complexes and intracellular signaling pathways, such as the endothelial protein C receptor (EPCR), protease-activated receptor1 (PAR1), and PAR3 [1, 2].

The PC gene (*PROC*) is located on chromosome 2 at position q14-21, comprising nine exons, one non-translated exon, and eight introns [3]. The presence of a short untranslated exon (exon 1) can affect the high-level and liver-specific expression of PC and produce different splice patterns for the human *PC* gene [4–7].

Numerous studies have reported that genetic polymorphisms in human PC are related to differences in PC activity and antigen levels, which are relevant to clinical characteristics and human pathophysiology [8–10]. Rare genetic polymorphisms in the human PC can cause PC deficiency states due to reduced secretion or synthesis of PC (Type I) or changed PC structure (Type II) [11]. On the other hand, common genetic polymorphisms have also been suggested to be associated with relative PC deficiency and with altered outcomes in disorders such as pulmonary embolism, venous thrombosis, cardiovascular disease, stroke, sepsis, and systemic meningococcemia [12–14]. Moreover, previous studies have demonstrated that the genetic variants of human PC contribute not only to diseases and disorders but also to the inter-individual variability in dose requirements for anticoagulant drugs such as warfarin. These genetic variations are linked to slightly lower concentrations and activities of PC, which influence the prothrombin time and result in lower warfarin dose requirements [15, 16]. Interpreting this information into the clinical guidelines on drug prescriptions could have a significant impact on the safety and efficacy of anticoagulant drugs.

The molecular mechanism of human PC mutations is associated with altered post-translational modification sites on PC, impaired folding and stability of the protein, changes in transcriptional regulatory regions, and reduced affinity for interaction with the metal ions or binding to the endothelial cell PC receptor. These alterations result in structural and functional changes in human PC [17–21].

Single nucleotide polymorphisms (SNPs) represent the most predominant types of genetic variations in the human genome and serve as biological markers to identify the genes associated with different Mendelian and complex genetic disorders [22]. Non-synonymous SNPs (nsSNPs), a nucleotide transition consistent with the amino acid change in the protein product, in particular missense SNPs, are primarily responsible for the phenotypic characteristics associated with various hereditary diseases because of their adverse effects on the structure, stability, charge, solubility, and function of the protein [23, 24]. Therefore, structural and functional analysis of nsSNPs may be instrumental in the development of precision medicine-based treatments and can assist in the design of effective and individualized drugs with fewer serious side effects for diseases caused by these genomic variations [25–27].

Currently, computational approaches have been extensively utilized to identify significant high-risk nsSNPs that have the potential to influence protein function [28]. Bioinformatics prediction tools provide advantages over experimental characterization because of their reliability, speed, convenience, and lower cost for evaluating whether changes are harmful [29]. Furthermore, analyses of the structure-function relationship using molecular dynamics (MD) simulations can ascertain the molecular mechanisms of diseases and provide valuable information for diagnosis and treatment [30].

Although few in silico studies on the functionality of human PC genetic variants are available [19, 31, 32], to the best of our knowledge, a comprehensive and systematic study that provides an in-depth and in silico analysis of the impact of all missense mutations on the structure and function of human PC is still required. Therefore, in the current study, a comprehensive investigation was performed to prioritize, characterize, and confirm the effects of each missense SNP on the human PC function. To achieve this, we employed multiple prediction algorithms to identify high-risk missense SNPs in human PC. Furthermore, MD simulations and molecular docking were applied to estimate the impact of mutations on protein structure and function more precisely. This work might help to determine the deleterious missense SNPs in human PC, which are responsible for the pathogenesis of diseases.

## Methods

### SNP dataset

The missense SNPs of the human *PC* gene were retrieved from the NCBI dbSNP database and mapped on genome assembly GRCh37.p13 (hg19) using Variation Viewer [33]. We utilized "*PROC*" as a keyword for search and filtered SNPs. (https://www.ncbi.nlm.nih.gov/variation/view/?q=PROC). Furthermore, we mapped these SNPs to the genomic coordinates of the "NM_000312.3" transcript expressing the human *PC* gene (UniProt ID: P04070) in our computational analysis [34]. Other databases such as ClinVar, OMIM, PharmGKB, and DisGeNET were also searched to cross-check the missense SNPs data for the human *PC* gene.

### Prediction of high-risk nsSNPs

Currently, various computational tools are being used to predict the functional and structural consequences of missense SNPs. In this step, bioinformatics tools were classified into two groups to determine the functional and structural impact of deleterious nsSNPs on the human PC. FATHMM‑MKL [35], PROVEAN [36], SIFT [37], Mutation Assessor [38], CADD [39], SNAP2 [40], Align GVGD [41], and PolyPhen2 [42] were used to predict the effect of the nsSNPs on the protein function. PhD-SNP [43], SNP and GO [44], SuSPect [45], Meta-SNP [46], PMUT [47], and VEST4 [48] were employed to predict the disease-related nsSNPs of the human *PC* gene.

### Prediction of high-risk nsSNPs position in the functional domains

The InterPro tool was used to determine the location of nsSNPs in different domains of the human PC structure [49]. InterPro is an integrated database that determines and recognizes results from various databases, including the Conserved Domains Database (CCD), SMART, Pfam, and Prosite.

### Conservation profile of high-risk nsSNPs

It was confirmed that nsSNPs positioned at highly conserved amino acid sites showed more deleterious effects than nsSNPs located at non-conversed positions [50, 51]. For this purpose, ConSurf, a web-based tool, was used to estimate the degree of evolution and conservation of human PC residues (using conservation scores) and determine their locations in the protein structure (buried or exposed) [52, 53]. The server computes the scale of conservation with a score between 1 and 9. A score from 1 to 4 is known as a variable, whereas a score between 5 and 6 is the average. A score of 7–9 was considered to indicate conservation.

### Prediction and evaluation of the 3D structure of human PC

As the tertiary structure of a human full-length PC does not exist in the PDB Bank, homology modeling using the Alpha Fold server was performed. This web server uses deep learning-based and machine-learning approaches to predict the protein structure [54]. The ProSA web server compared the quality of homology-modeled protein structures by proteins, and their structures were revealed by X-ray and NMR methods [55]. The quality of homology modeling was investigated using web servers such as Procheck [56], Varify3D [57], ERRAT [58], and MolProbity [59]. The Procheck web server qualified homology modeling based on the Ramachandran plot. Varify3D web server compared the compatibility of 1D protein structure with its 3D structure. Verify3D values greater than 80% indicated high accordance between the 1D and 3D protein structures. The ERRAT web server checks the statistics of non-bonded interactions between different atom types. An ERRAT value of more than 95% indicates that the protein structure quality is comparable to that of the X-ray and NMR structures. MolProbity calculates the Clash score, rotamer, and Ramachandran estimations into a single score, which is identified as the MolProbity score. The initial structure of the high-risk SNPs of human PC was determined using Chimera software with Dunbrack Library 2010 [60]. Energy minimization was performed using the Amber force field for mutant forms that had a clash score during mutation compared to the native form. The parameter of energy minimization was 100 steps, and the steepest descent algorithm was applied with the size of 0.02 angstroms and 10 conjugated gradient steps.

### Predicting effects of high-risk nsSNPs on protein stability

The essential feature that influences the function, activity, and regulation of biological molecules is protein stability [61]. The protein-free energy is a crucial index of protein stability [62]. The effect of mutations on protein stability can be precisely established by evaluating the impact of high-risk nsSNPs on free energy. To do this, the stability changes of high-risk nsSNPs in human PC were analyzed using sequence-based tools like I-Mutant 2.0 [63], MUpro [64], and INPS-MD [65], and structure-based algorithms such as DynaMut [66], CUPSAT [67], mCSM [68], and DUET [69].

### Molecular dynamic simulation analysis

MD simulations were performed using GROMACS version 2020.3, to study the effect of each SNP on protein structure compared to its native form [70]. Each structure was located in a 10 A° cubic simulation box filled with the simple point charge (SPC) water molecule type. The optimized potential for liquid simulations (OPLS) force field was applied to simulate all the structures. Na^+^ and Cl^-^ were used to neutralize the charge of the simulation system. For the minimization simulation systems, the steepest descent minimization integrator was executed for 5000 minimization steps until the maximum energy of the simulation systems reached less than 1000 KJ.mol^-1^ nm^-1^. The simulation systems were then equilibrated with NVT (constant number of particles, volume, and temperature) by velocity-rescaling (V-rescale) as a modified Berendsen thermostat at 300 K for 100 picoseconds (ps). NPT (constant number of particles, pressure, and temperature) equilibration was performed with Parrinello-Rahman at 1 bar for 100 ps. Then, the equilibrated system was entered into MD simulations with a time step of two femtoseconds (fs) and 200 nanoseconds (ns) of the simulation run. The linear constraint solver (LINCS) algorithm was used to recognize the constraints for all bonds. The non-bonded electrostatic interactions for long-range electrostatic interactions were calculated using the particle mesh Ewald (PME) algorithm. The cut-off for both long electrostatic and van der Waals interactions was set at 1.0 nm. Periodic boundary conditions were considered for all the simulations.

The root mean square deviation (RMSD), the radius of gyration (Rg), the root mean square fluctuation (RMSF), and the solvent accessible surface area (SASA) were analyzed using gmx rms, gmx gyrate, gmx rmsf, and gmx sasa with the donor-acceptor set at a maximum of 0.35 nm in GROMACS package, respectively. The secondary structure content of the proteins was calculated as a function of time using the DSSP program to figure out the stability and structural changes in each simulation system during the simulation. Principal component analysis (PCA) was conducted using the gmx cover to determine the motions of the residues related to a set of linearly unrelated variables. For PCA, the first two eigenvalues and eigenvectors, according to the carbon alpha atom motion, were used to compute the covariance matrix. The eigenvectors are related to the direction of motion, while the eigenvalues represent the degree of motion along the direction. The free energy landscape (FEL) shows the possible conformations of proteins during simulation based on Gibbs free energy.

### Predicting effects of high-risk nsSNPs on protein-protein interaction

The effect of high-risk nsSNPs on the interaction with coagulation factor V (FV), which plays a critical role in the coagulation pathway, was analyzed by molecular docking using the HADDOCK tool [71] and ClusPro web server [72]. To do this, the native form of human PC with the active form of Factor V protein (FVa) was docked along with the mutant models of PC by defining active residues of the binding site and considering default molecular docking restraining parameters in the HADDOCK and ClusPro web servers. The restraint parameters are automatically turned on in HADDOCK, consisting of ambiguous interaction restraints (AIRs) to identify protein interfaces, surface contact restraints to define an ambiguous distance between two molecules, and center of mass restraints to ensure that two molecules are in contact. The ClusPro web server applied the PIPER docking algorithm for protein-protein docking. The 3D structure of FVa was retrieved from the PDB Bank (PDB ID:7kxy). The binding affinity of each complex was characterized using the PRODIGY web server [73]. Molecular docking results were analyzed using the Ligplot package. Furthermore, the effect of each mutation on the protein-protein interaction of human PC with FVa was assessed using the Mutabind2 [74] and mCSM-PPI2 [75] web servers.

## Results

### SNP annotation

**Fig 1:**
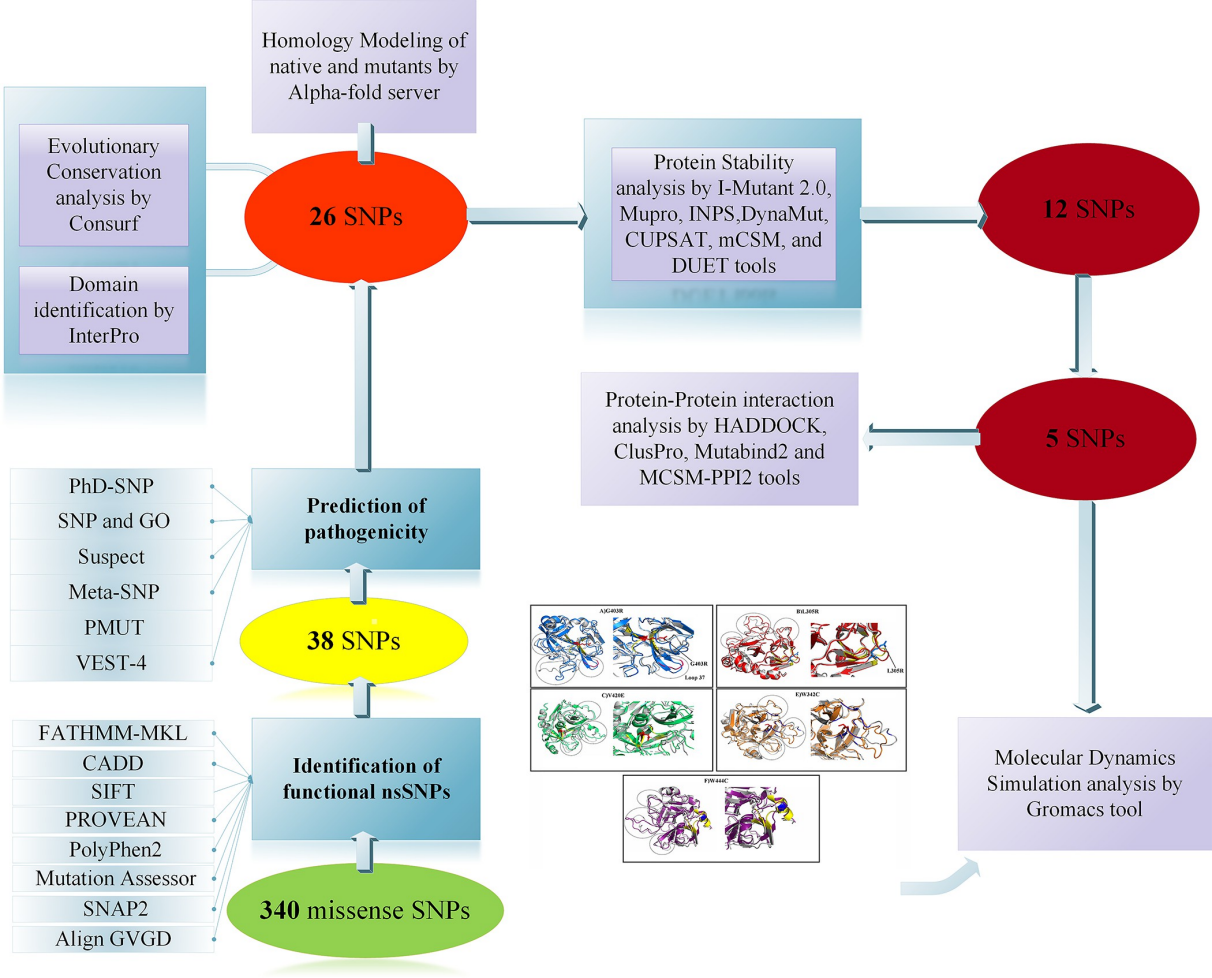
A flowchart describes the stepwise analysis of missense SNPs in the human PC and illustrates the computational tools used in the study. A total of 340 missense SNPs were selected for this investigation. In the first step, 38 SNPs were discovered to be related to functional effects by utilizing eight tools. Among them, 26 missense SNPs were found as pathogenic SNPs. Eventually, 12 mutations were predicted as the most high-risk missense SNPs. The five mutants L305R, W342C, G403R, V420E, and W444C were subjected to MD simulations. Furthermore, the effects of these five mutations on human PC-FVa interaction were detected by HADDOCK, ClusPro, MutaBind2, and mCSM-PPI2 tools.

We retrieved SNPs of human PC using the NCBI dbSNP database, which contained 2,266 SNPs in the intronic region, 37 SNPs in the 5′UTR region, 73 SNPs in the 3′UTR region, and 360 SNPs were nsSNPs (340 Missense and 20 Nonsense). Only missense SNPs of the human *PC* gene were selected for this investigation due to their direct participation in disease pathogenesis and their effect on the adopted treatment regimen. The overview of the methodological approach is summarized in a schematic diagram (Fig 1).

### Predicting deleterious nsSNPs

Computational analysis was performed using various tools in a multi-step framework to identify functional SNPs among the 340 missense SNPs of the human *PC* gene. All missense SNPs were evaluated for initial screening using FATHMM‑MKL, PROVEAN, SIFT, Mutation Assessor, CADD, SNAP2, Align GVGD, and PolyPhen2. The results illustrated that 38 nsSNPs were associated with functional effects on human PC using all methods (S1 Table). Subsequently, all 38 nsSNPs of the human *PC* gene were further analyzed for correlation with the disease after functional impact through PhD-SNP, SNP and GO, Suspect, Meta-SNP, PMUT, and VEST4 (S2 Table). Eventually, 26 nSNPs out of the 38 examined nsSNPs of the human *PC* gene were classified as disease-related nsSNPs by all six different methods and selected as "high-risk missense SNPs" for further analysis (Table 1).

**Table 1:** List of " 26 high-risk missense SNPs of human PC" identified by six *in silico* programs.

SNP ID	Substitution	PhD-SNP	SNP and GO	SuSPect	Meta-SNP	PMUT	VEST4
**rs774572099**	R42S	Disease	Disease	Disease	Disease	Disease	Disease
	R42C	Disease	Disease	Disease	Disease	Disease	Disease
**rs757583846**	R57W	Disease	Disease	Disease	Disease	Disease	Disease
**rs574949343**	R57L	Disease	Disease	Disease	Disease	Disease	Disease
**rs121918148**	E62A	Disease	Disease	Disease	Disease	Disease	Disease
**rs1448630830**	E67K	Disease	Disease	Disease	Disease	Disease	Disease
**rs1171885932**	C111Y	Disease	Disease	Disease	Disease	Disease	Disease
**rs747735192**	C140S	Disease	Disease	Disease	Disease	Disease	Disease
	C140F	Disease	Disease	Disease	Disease	Disease	Disease
**rs1247269491**	C147Y	Disease	Disease	Disease	Disease	Disease	Disease
**rs767201513**	C151R	Disease	Disease	Disease	Disease	Disease	Disease
**rs199469474**	C175Y	Disease	Disease	Disease	Disease	Disease	Disease
**rs1277271891**	W225R	Disease	Disease	Disease	Disease	Disease	Disease
**rs757925208**	W225C	Disease	Disease	Disease	Disease	Disease	Disease
**rs748099849**	C238S	Disease	Disease	Disease	Disease	Disease	Disease
**rs777486993**	G239R	Disease	Disease	Disease	Disease	Disease	Disease
**rs749500010**	G239E	Disease	Disease	Disease	Disease	Disease	Disease
**rs774584131**	I243T	Disease	Disease	Disease	Disease	Disease	Disease
**rs375156587**	L249P	Disease	Disease	Disease	Disease	Disease	Disease
**rs1353816203**	H253P	Disease	Disease	Disease	Disease	Disease	Disease
**rs1573459781**	L303R	Disease	Disease	Disease	Disease	Disease	Disease
**rs1241074486**	L305R	Disease	Disease	Disease	Disease	Disease	Disease
**rs781097228**	W342C	Disease	Disease	Disease	Disease	Disease	Disease
**rs1439742162**	C398R	Disease	Disease	Disease	Disease	Disease	Disease
**rs1442363621**	G403R	Disease	Disease	Disease	Disease	Disease	Disease
**rs760579201**	V420E	Disease	Disease	Disease	Disease	Disease	Disease
**rs1271213613**	Y435H	Disease	Disease	Disease	Disease	Disease	Disease
**rs121918142**	W444C	Disease	Disease	Disease	Disease	Disease	Disease

### Prediction of high-risk nsSNPs positions in the functional domains

The function of proteins is related to their domains, which are usually located in the highly conserved region of the protein, and any changes in these domains cause structural and functional variations in proteins. To find out the position of 26 high-risk missense SNPs in functional and conserved regions in human PC, the InterPro tool was applied.

**Fig 2:**
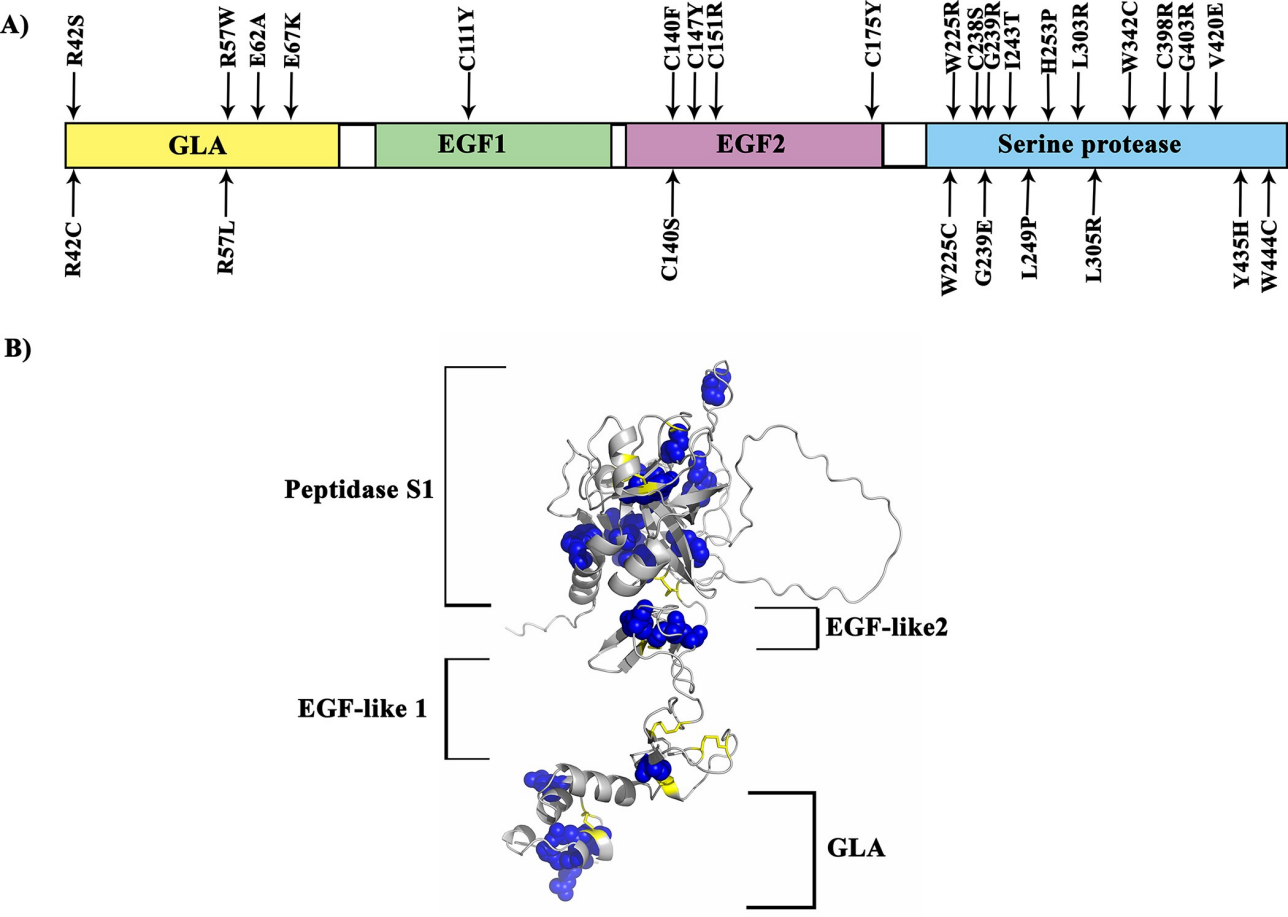
Domains and position of high-risk missense SNPs within the human PC. (A) The position of 26 high-risk missense SNPs (23 positions) on the four functional domains of human PC. (B) shows the position of 26 high-risk missense SNPs (23 positions) on the superimposed structure of four functional domains of human PC. As shown in this figure, most of the high-risk missense SNPs (16 SNPs) are located in the SP domain of human PC.

The InterPro tool predicted that four major domains were located in the human PC. These domains are the Gamma-carboxy glutamic acid-rich (GLA) domain (residues 24 to 88), EGF-like calcium-binding (EGF1) domain (residues 88 to 132), EGF-like (EGF2) domain (residues 97 to 176), and serine proteases- trypsin (SP) domain (residues 211 to 450). Moreover, there are two active site pockets in the SP domain, where the triad residues are accommodated (His253, Asp299, and Ser402). According to InterPro, all 26 "high-risk missense SNPs" that caused 28 mutant amino acid changes were positioned in the four functional domains of human PC. Six high-risk missense SNPs (R42S, R42C, R57W, R57L, E62A, and E67K) were presented in the GLA domain. This domain has a γ-carboxylation site in the post-translational process and is responsible for interaction with membrane surfaces. One missense SNP (C111Y) was located in the EGF1 domain, and five missense SNPs (C140S, C140F, C147Y, C151R, and C175Y) were located in the EGF2 domain. These two domains are responsible for interactions with other proteins that enhance the anticoagulant activity of PC. The rest 16 high-risk missense SNPs (W225R, W225C, C238S, G239R, G239E, I243T, L249P, H253P, L303R, L305R, W342C, C398R, G403R, V420E, Y435H, and W444C) were presented in the SP domain, which is essential for activating the PC. As shown in Fig 2, the highest number of high-risk SNPs were found in the SP domain, which could be due to the critical function of the SP domain in human PC.

### Conservation profile of high-risk nsSNPs

According to evolutionary studies, conserved sites in protein sequences are vital, and any change in these sites affects the structure and functional modifications of proteins [50, 51]. To determine whether 26 high-risk missense SNPs were located in the conserved region of the protein, the Consurf server was used (S1 Fig). Data showed that most of the high‐risk nsSNPs were highly conserved (Conservation Score 9). Among the highly conserved amino acid residues (20 positions), eight residues (R42, R57, E62, E67, C147, H253, W342, and G403R) were predicted to be functional and exposed, whereas 12 wild-type amino acids (C111, C140, C151, C175, W225, C238, G239, L305, C398, V420, Y435, and W444) were structurally and buried. Additionally, the L249 residue is predicted to be conserved on average (Score 6) and two remaining residues I243 and L303 are conserved (Score 7).

### Prediction and evaluation of the 3D structure of human PC

As the tertiary structure of human full-length PC did not exist in the PDB bank, and some of the mutations were located in regions where the tertiary structure was not accessible, homology modeling was applied (S2 Fig). The Alpha-Fold web server modeled the human PC. The results indicated that this protein comprised approximately 13% alpha-helix, 32% beta-sheet, and 53% coil structures with ten disulfide bonds (S3A Fig). Different model validation web servers assessed the quality of homology modeling (S3 Table). The ProSA web server compared the quality of the tertiary structure of human PC by proteins with a similar length and their structures were discovered through X-ray and NMR using the ProSA web server. The results indicated that the PC model Z-score was located in the zone where the protein structure characterized by X-ray and NMR was located (S3B Fig), indicating the high quality of the PC model. In addition, human PC structure analysis based on the Ramachandran plot using the Procheck web server revealed that more than 80% of amino acids are located in allowed and generally allowed regions that designate the good stereochemical quality of this protein structure. The Verify3D web server was used to investigate the compatibility between the primary and tertiary structures of the protein. The Verify 3D value of the PC was 79.71%, near 80%, showing high compatibility between the 1D and 3D human PC models. ERRAT expresses the quality of non-bond interactions in the protein structure. An ERRAT value of more than 95% represents a high-quality resolution of the protein structure. The ERRAT value of PC was 96.31%, indicating high protein structure quality in non-bonded interactions. The MolProbity score assesses the quality of the protein structure by characterizing the clash score, rotamer, and Ramachandran evaluation into a single normalized score, which is equivalent to the X-ray resolution scale. The MolProbity score of the human PC was 1.09, which showed high protein structure quality compared to the X-ray resolution scale. All the validation results indicated that the human PC model had high quality; hence, this structure was used for further analysis in this study.

### Predicting effects of high-risk nsSNPs on protein stability

Protein stability is the net balance of forces that determines whether a protein is in the natively folded form or denatured. To predict the effects of amino acid substitutions caused by nsSNPs on protein stability, online tools such as I-Mutant 2.0, MUpro, INPS-MD, DynaMut, CUPSAT, mCSM, and DUET, were applied based on the sequence or structure of the protein. These tools assessed the stability of proteins based on protein-free energy value (DDG) changes in the mutant form compared to the wild type. Among the 28 missense amino acid changes in the human PC, I-Mutant 2.0, MUpro, INPS-MD, DynaMut, CUPSAT, mCSM, and DUET predicted that 22, 28, 26, 18, 23, 26, and 23 mutant amino acids exerted a decreasing DDG value (DDG < 0), respectively. Out of all, 12 mutations (R42S, W225C, G239R, I243T, L249P, L303R, L305R, W342C, G403R, V420E, Y435H, and W444C) were predicted to be destabilizing (DDG < 0) using all seven software tools (S4 Table). Reduced protein stability leads to increased aggregation, aberrant conglomeration, and protein misfolding, resulting in protein malfunction. Hence, these 12 high-risk missense SNPs may cause maximum damage to protein function by affecting stability. For this reason, we considered these 12 high-risk missense SNPs as "the most high-risk missense SNPs" of human PC.

### Molecular dynamic simulation analysis

To explore which SNPs should be subjected to MD simulations, we normalized the damaging scores of the most high-risk missense SNPs (12 SNPs) that were predicted by all tools based on linear min-max scaling (S5 Table). This approach showed that different damage scores had a distribution range of 0 and 1 (S6 Table). The five most high-risk missense SNPs (L305R, W342C, G403R, V420E, and W444C) with the highest scores were selected for MD simulations.

The MD simulation approach is broadly used as a valuable tool for distinguishing disease-related SNPs from neutral SNPs. MD simulations were applied to determine the effect of each mutation on protein tertiary structure changes under natural simulation conditions. The most high-risk missense SNPs, including L305R, W342C, G403R, V420E, and W444C, are located in the SP domain, which is the functional domain of human PC. Therefore, only the structure of the SP domain was subjected to MD simulations to explore the possible impacts of each substitution on protein structure.

The MD simulation was performed twice for 200 ns using GROMACS software to reproduce the simulation data. We considered a comparative analysis of the average results of each simulation system for the native and five mutant proteins (S7 Table). The number of solvents and ions for each simulation system is represented in the S8 Table.

**Fig 3:**
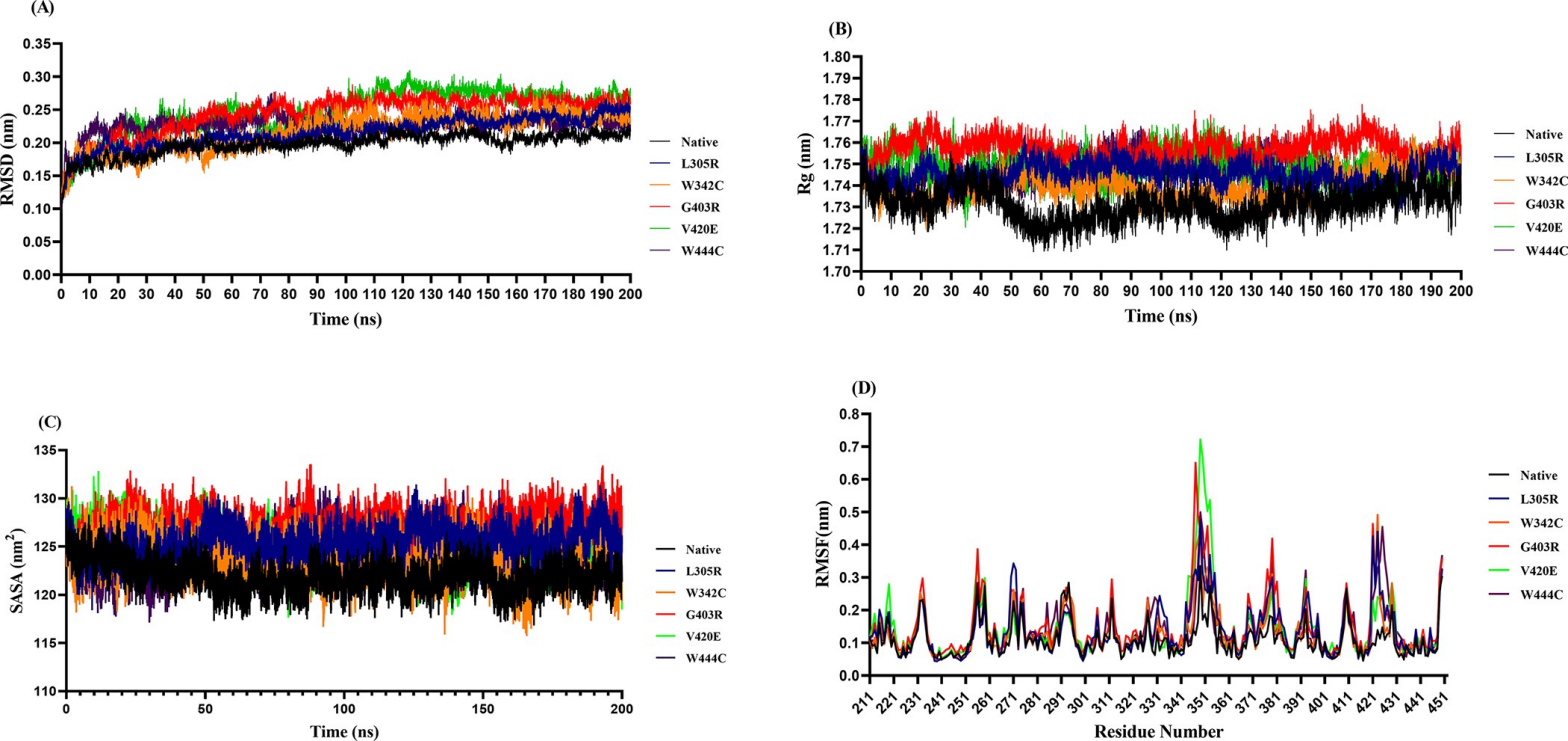
Analysis of MD simulations for the SP domain of native PC and its mutant structures (L305R, W342C, G403R, V420E, and W444C) over 200 ns simulations. (A) the backbone RMSD plot for the SP domain of native PC and its mutant models. (B) The Rg plot for the SP domain of native PC and its mutant models during the entire simulation time. (C) The solvent accessible surface area (SASA) plot for the SP domain of native PC and its mutant models. (D) the RMSF plots of the Cα atoms as a function of residue number for the SP domain of native PC and its mutant models during the entire simulation time. The color scheme is as follows: native PC (black color), L305R mutant (blue color), W342C mutant (orange color), G403R mutant (red color), V420E mutant (green color), and W444C mutant (purple color).

RMSD denotes the conformational stability of macromolecules during the simulation [76]. The RMSD value analysis among native and mutant structures exhibited deviations between ~0.06 nm and ~0.27 nm and reached a state of equilibrium after 50 ns (Fig 3A). Our analysis revealed that, out of all six trajectories, the native protein showed the least deviation pattern, with a stable conformation at 50 ns and converging at approximately 0.19 nm. Among the five mutations, L305R and W342C mutants demonstrated the least deviation pattern and converged at an around RMSD value of 0.21 nm (Fig 3A). Meanwhile, the G403R and V420E mutants exhibited the most substantial deviating pattern, converging with an RMSD value of around 0.24 nm (Fig 3A). Finally, W444C mutant displayed a maximum deviation of around 0.27 nm and converging with an RMSD value of approximately 0.22 nm (Fig 3A). These differences in the deviation range between the native and mutant proteins elucidate the effect of mutations on protein structure, thereby providing a basis for subsequent analyses.

Rg analysis provides complete insight into protein compactness and relaxation during MD simulations by calculating the root mean square distance of a group of atoms from the axis of rotation. Higher Rg values indicate that the protein structure is less compact and more flexible. As illustrated in Fig 3B, the lowest compactness was observed in the G403R mutant, with an Rg value of approximately 1.76 nm, whereas the native protein had the lowest Rg value of approximately 1.72 nm. The L305R, W342C, V420E, and W444C mutants showed greater Rg values (~ 1.74 nm) over time when compared to the native protein (Fig 3B). The results predicted that all five mutants affected the compactness of the human PC structure and changed the conformation of the protein to a more unstable form.

SASA shows the surface area of a biomolecule that is accessible to a solvent [77]. This parameter is crucial for identifying the stability and folding of protein structures. SASA plot analysis illustrated that all mutants had more fluctuations than their native form (Fig 3C). The SASA values revealed that among all five mutants, the G403R mutant displayed a much higher SASA value (∼127.4 nm2) than the native form (∼121.5 nm2). Meanwhile, the W342C mutant exhibited a slightly higher SASA value (∼123.5 nm2) than the native protein. The SASA values for the L305R, V420E, and W444C mutants were approximately 125.75 nm^2^, 124.15 nm^2^, and 124.70 nm^2^ respectively. These differences in SASA values in mutant proteins indicated lower overall structural stability and folding of human PC due to the five mutations.

The RMSF evaluation based on residue displacement identifies the local flexibility, thermal stability, and heterogeneity of macromolecules during MD simulations [67]. The RMSF plot showed a higher fluctuation in the mutant structures compared to the native form (Fig 3D). The RMSF value for the native protein was approximately 0.11 nm. For the L305R, W342C, G403R, V420E, and W444C mutants, RMSF values were approximately 0.14, 0.13, 0.15, 0.14, and 0.14 nm, respectively. Hence, all mutants showed higher fluctuations than the native protein, suggesting that the mutations affected the overall conformational flexibility of human PC.

To analyze the effect of each mutation on protein structure, the RMSF plot of each mutation was aligned to the RMSF plot of the native protein (S4 Fig). Assessment of the RMSF plots revealed that all mutations (L305R, W342C, G403R, V420E, and W444C) increased the fluctuation of residues in the autolysis loop (residues 344–359) and residues 420–430 near the Na^+^ binding loop in the SP domain compared to the native form (S4 Fig). Furthermore, the G403R mutant showed a greater fluctuation of residues 371–382 in the SP domain compared to the native protein (S4 Fig).

**Fig 4:**
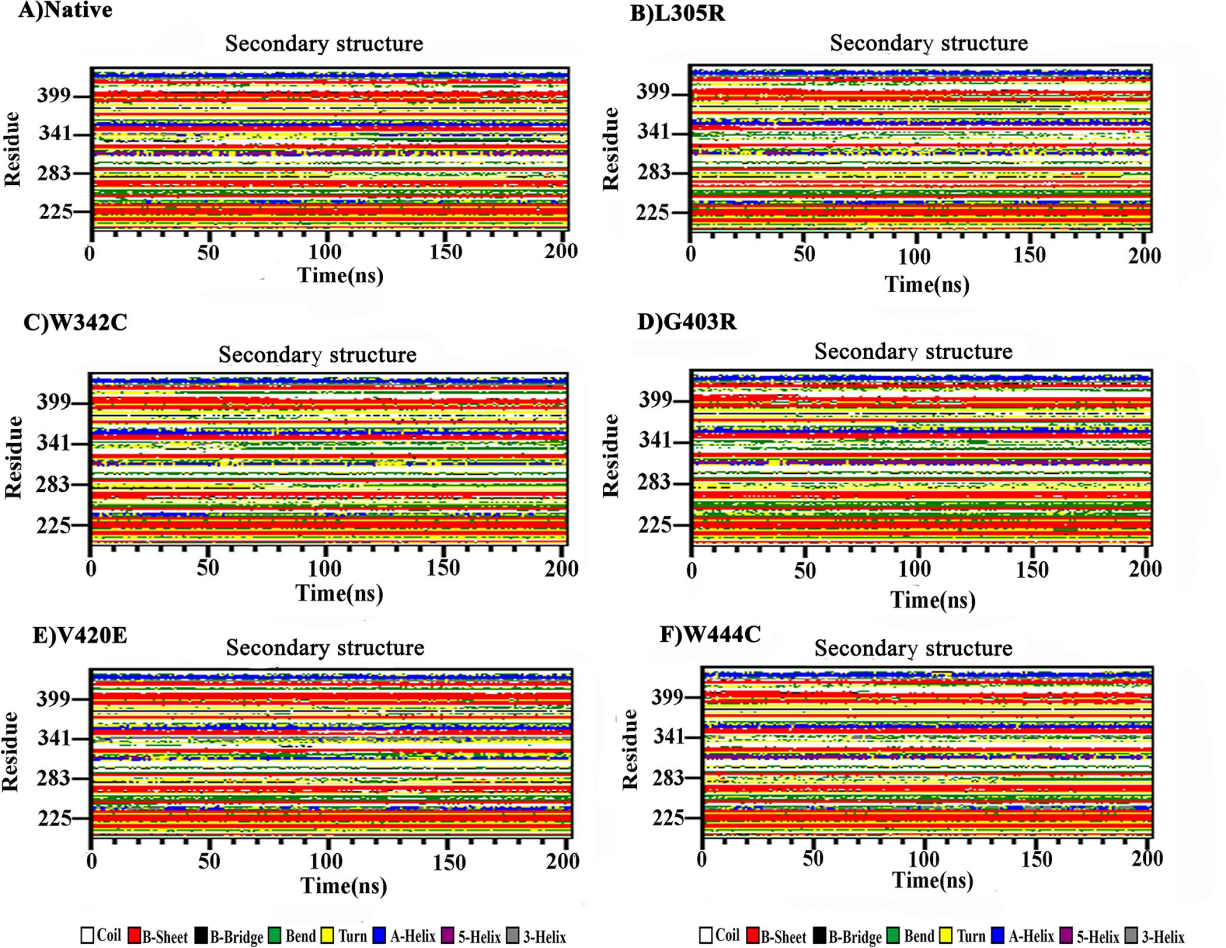
Secondary structure profile analysis for the SP domain of native PC and its mutant structures over 200 ns MD simulation times. (A) Native PC, (B) L305R mutant, (C) W342C mutant, (D) G403R mutant, (E) V420E mutant and (F) W444C mutant.

Secondary structure element (SSE) analysis revealed that each mutation caused a slight change in the secondary structure during the simulation (Fig 4). However, SSE analysis showed that all mutants induced a slightly more coiled conformation in residues 230–357, whereas they disrupted the β-sheet conformation in residues 380–420 of the SP domain compared to the native form. The other elements were marginally similar to those of the native protein.

The assessment of the percentage of each state’s secondary structure in protein structures revealed that a slight decrease in the secondary structure content could be seen in the case of all mutations (L305R, W342C, G403R, V420E, and W444C) compared to the native form, which caused more structural flexibility in mutant forms than in the native protein (S9 Table). Considerable changes in the secondary structure profile of all mutants were found due to coil formation and loss of β-sheet compared to the native form (S9 Table).

**Fig 5:**
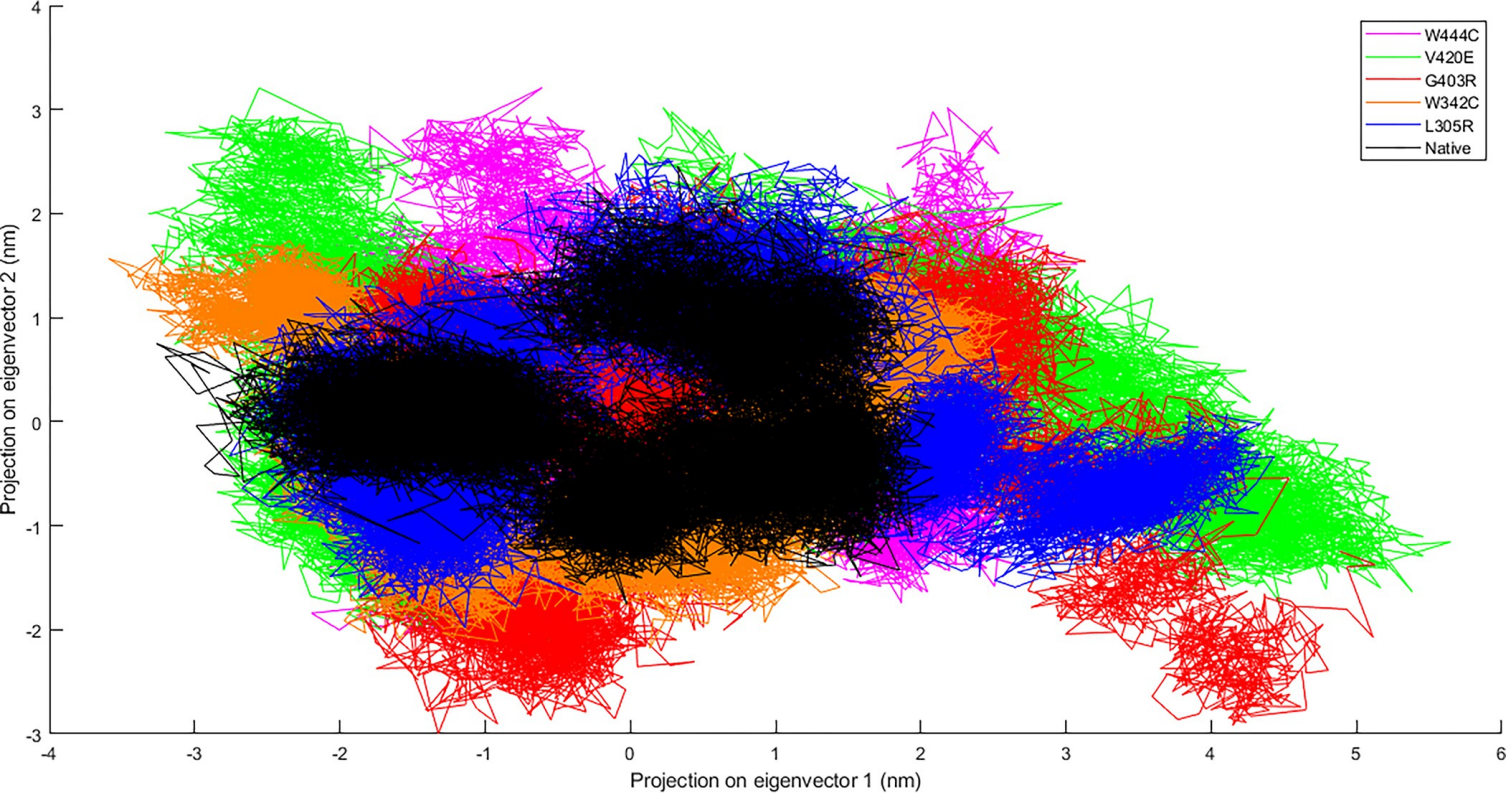
Principal component analysis (PCA) for the SP domain of native PC and its mutant structures over 200 ns MD simulation times. Comparison of L305R mutant (blue color), W342C mutant (orange color), G403R mutant (red color), V420E mutant (green color), and W444C mutant (purple color) with native form (black).

PCA was performed to examine whether these substitutions affected the overall conformation and flexibility of the protein during the simulation. The observation of the corresponding eigenvalues showed the level of variation and dynamic nature of the protein molecules in the simulation system, which was mostly restricted to the first two eigenvectors. The PCA plot indicated that all mutants had a more expanded structure and a broader range of eigenvectors compared to the native form (Fig 5). Furthermore, the results showed that the G403R and V420E mutants had the greatest increase in conformational space compared to the native protein and all other mutant models (Fig 5).

The PCA results are compatible with the trace of covariance matrix values after diagonalization, which showed that L305R, W342C, G403R, V420E, and W444C mutants had covariance matrix values of approximately 9.39 nm^2^, 7.87 nm^2^, 10.35 nm^2^, 11.01 nm^2^, and 7.57 nm^2^, respectively. Meanwhile, the covariance matrix value of the native form was 5.47 nm^2^, which indicated more flexibility for all five mutants compared to the native structure.

**Fig 6:**
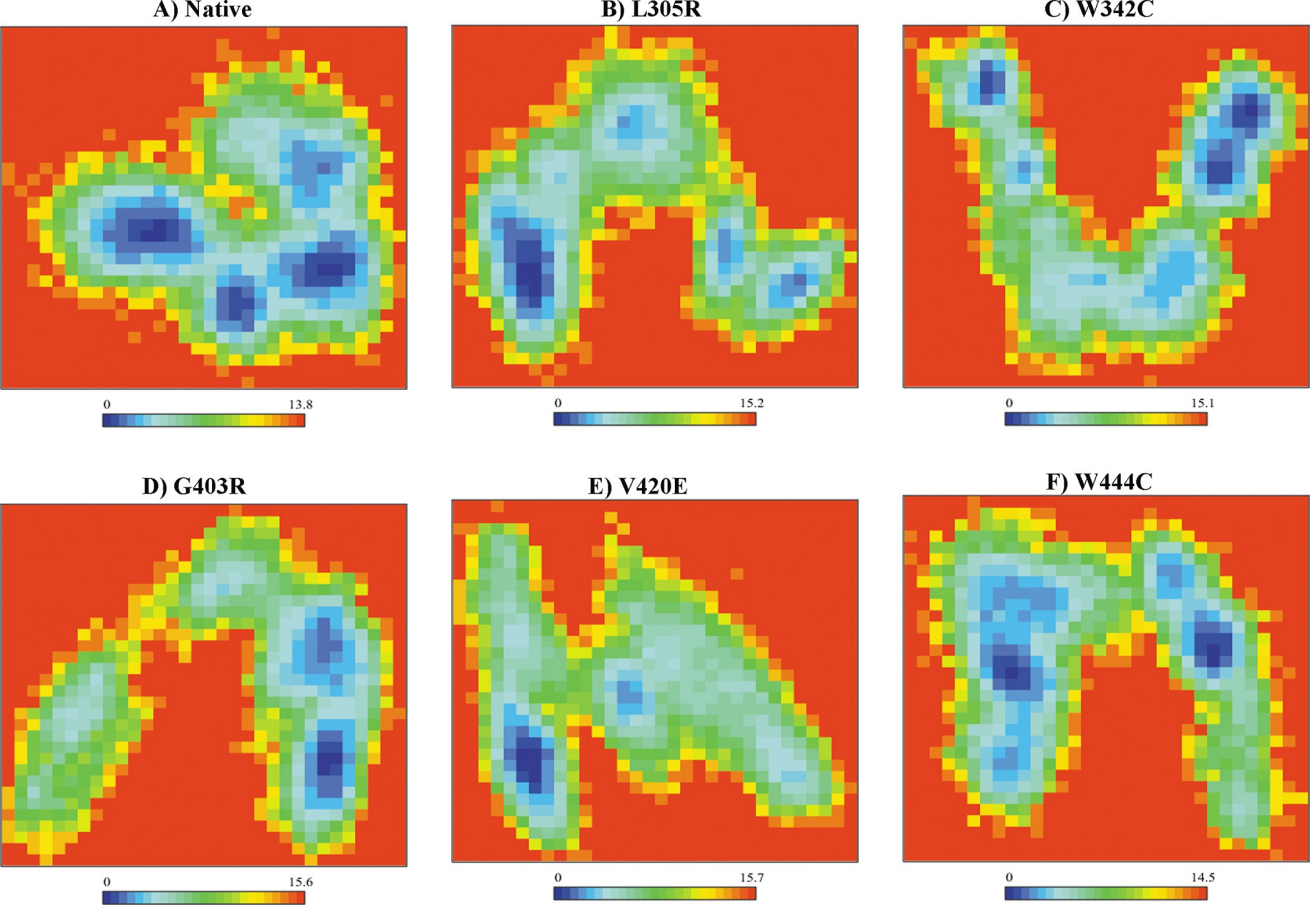
Free energy landscape (FEL) analysis for the SP domain of native PC and its mutant structures over 200 ns MD simulation times. (A) Native PC, (B) L305R mutant, (C) W342C mutant, (D) G403R mutant, (E) V420E mutant and (F) W444C mutant.

FEL was applied to more accurately depict the conformational behavior of the native PC structure and its mutant models. In the FEL analysis, the energy minima and energetically preferred protein conformations are shown by blue spots, whereas unfavorable conformations are represented by red spots. As shown in Fig 6, the native structure seemed more stable based on its size and the minimum energy region (blue spots) compared to all mutant models. Furthermore, L305R, W342C, G403R, V420E, and W444C mutants exhibited a greater range of Gibbs’s free energy value compared to the native form, especially for G403R and V420E mutations with the Gibbs’s free energy value ranges from 0 to 15.6 kJ/mol and 0 to 15.7 kJ/mol, respectively. Because the folding pattern of the protein directly affects its stability, more unfolded states suggest the destabilization of human PC in the case of all five mutants.

### Predicting effects of high-risk nsSNPs on protein-protein interaction

The high-risk nsSNPs in proteins have shown that changes in molecular interactions and substrate specificity can cause different diseases and disorders. The five most high-risk missense SNPs (L305R, W342C, G403R, V420E, and W444C) are located in the SP domain of human PC, we hypothesized that these mutations could influence the interaction between PC and its substrate FVa. To support our hypothesis, we used computational docking web servers to perform molecular docking studies on native and mutant PC models with FVa. Due to the lack of the crystal structure of the APC-Fva complex in the PDB bank, two docking web servers (HADDOCK and ClusPro) were applied to validate the docking results. The binding site of PC was identified according to a previous study, which interacts with Arg 506 and Arg 306 as two critical amino acids in FVa that cleave FVa to the inactive form [78]. The best docking pose of each mutation in both web servers was selected for analysis using the Ligplot package (S10 Table). The data showed that all mutants had a lower binding affinity (KD) compared to the native structure, except for the W444C mutant. In addition, the effect of each mutation on the interaction with Fva was analyzed using the mcSM-PPI and Mutabind2 tools (S10 Table), which revealed that all mutants decreased their affinity compared to the native form and had a harmful effect on the PC-Fva interaction. The RMSD value of the structural alignment was less than 0.5, indicating the same conformation between the two structures. The RMSD value of each PC-Fva complex revealed that the PC-Fva complex results of each docking web server had the same conformation (S10 Table).

## Discussion

The *PC* gene encodes PC, a vitamin K-dependent glycoprotein that performs an important function in controlling anticoagulant processes and acts as a cytoprotective agent in cell survival and apoptosis. Genetic variations in the *PC* gene have also been reported to be related to several diseases. However, only a few in silico studies on the functionality of *PC* genetic variants are available [19, 31, 32]. This study is a comprehensive and systematic investigation based on a computational approach to figure out the most deleterious SNPs in the *PC* gene. Hence, for this purpose, we have exploited the computational approach by utilizing various in silico tools with different algorithms for the complete analysis of missense SNPs in the *PC* gene to figure out the most deleterious SNPs that could influence the activity of human PC.

Among the 340 missense SNPs found in the *PC* gene, we finally screened 26 significantly deleterious missense SNPs using all fourteen prediction tools as “high-risk missense SNPs”. Analysis based on the InterPro tool determined that these 26 high-risk missense SNPs were positioned in the four functional domains of human PC, of which 16 SNPs were found in the SP domain (Fig 2). So, this region of the protein can be regarded as a mutationally sensitive region of human PC.

The SP domain, known as the catalytic domain of human PC, contains a His active site pocket (residues 249–254) and a Ser active site pocket (residues 396–407) with three catalytic residues, His 253, Asp 299, and Ser 402. Moreover, this domain includes some functional binding loops, including loop 37 (residues 232–235), loop 60 (residues 256–264), loop 70 or Ca^2+^-binding loop (residues 267–277), loop 148 or autolysis loop (residues 344–359) and Na^+^ binding loops (residues 388–396 and 427–432), which these loops create a positively charged basic area on the surface of PC for binding substrates before cleavage of human PC. Therefore, any amino acid substitution in the SP domain may alter the binding of PC with its substrates, which affects protein function [79–81]

Evolutionary conservation analysis indicated that most of the high-risk missense SNPs occupied conserved residue positions. Therefore, any change in these amino acids can affect the regulation of the biological function of the protein. These data are consistent with previous experimental studies that found that the mutants R42S [82], R57W [83, 84], E62A [85], V420L [86], and W444C [87] are associated with thrombophilia disorders due to PC deficiency.

Protein stability analysis of 26 high-risk missense SNPs illustrated those 12 mutations (R42S, W225C, G239R, I243T, L249P, L303R, L305R, W342C, G403R, V420E, Y435H, and W444C) lead to a decrease in protein stability. Typically, the regulation, activity, and function of a protein, significantly are determined by its structure stability. Therefore, these 12 high-risk missense SNPs might have the maximum damage potential to abolish the structure and function of human PC. This data is compatible with the experimental findings by W Tsay et al. which revealed that the substitution of the Ile to Thr in position 243 (I243T) caused abnormal folding of human PC and was associated with type I PC deficiency and venous thrombosis disorder [88].

After analysis of human PC mutations by different computational web servers, data showed that L305R, G403R, V420E, W342C, and W444C mutants were the most deleterious mutations in human PC, therefore, the effects of these high-risk missense SNPs on protein stability and dynamic behavior at the atomic level were analyzed using MD simulations. As demonstrated by Yun et al., a greater RMSD was associated with reduced stability [89], which was consistent with this study that the G403R and V420E mutant models showed the largest deviation pattern among the five mutant structures, suggesting that these two mutations had a negligible impact on protein stability. In addition, the L305R, W342C, and W444C mutants showed a higher degree of RMSD fluctuation than the native form, indicating that these mutations may lead to unstable structures under physiological conditions (Fig 3A).

Conformational alterations are essential for the physiological function of proteins. However, there must be a fine balance between conformational rigidity and flexibility. Therefore, based on the results obtained from Rg analysis, the L305R, W342C, G403R, V420E, and W444C mutants showed a clear difference in the compactness of human PC compared to the native structure (Fig 3B). Consequently, greater flexibility and lower overall compactness may have harmful effects. In support of this finding, SASA analysis showed that the high flexibility induced by all five mutant structures (Fig 3C), especially the G403R mutant, could potentially lead to the expansion of the surface area exposed to the solvent, resulting in protein misfolding.

From the RMSF analysis (Fig 3D), we observed changes in flexibility in all five mutant models (L305R, W342C, G403R, V420E, and W444C). In particular, mutant models showed high fluctuations in residues 344–359 of the autolysis loop and residues near the Na^+^-binding loop in the SP domain compared to the native form (S4 Fig). A study by Gale et al., revealed that any changes in human PC conformation, especially in the loops 37, 60, 70, and autolysis loop, could affect the interaction of PC with Fva and lead to the loss of proper PC function [78]. Consistent with the current results, Romeo et al. demonstrated that the W444C mutant reduced human PC activity and caused a type II PC deficiency disorder [87]. Furthermore, more local residue flexibility was observed in the G403R mutant among the five mutant models compared to the native protein. As Gly 403 is localized near the catalytic residue (Ser 402), any mutation in this region could affect the proper active site geometry and flexibility of the protein. In addition, the W342C mutant is near the Asp269 and Leu 270 residues in loop 70 (Ca^2+^-binding loop), which is responsible for interaction with Fva.

Gaining insight into alterations within the secondary structural arrangement of a protein can offer valuable insights into its folding mechanism and conformational behavior. The results obtained from the SSE analysis provided evidence of a slightly more coiled conformation and lower B-sheet formation in all five mutant models (L305R, W342C, G403R, V420E, and W444C) compared to the native protein (Fig 4). The transition from the β-sheet to coil conformation may lead to reduced protein stability because random coils lack distinct secondary elements, potentially affecting protein function [90, 91].

Proteins exhibit coordinated motions between atoms to perform distinct functions. Based on the PCA (Fig 5) and FEL results (Fig 6), the five mutant models showed greater collective motion than the native protein, and the G403R and V420E mutants showed the most highly dispersed motion among all five mutant models. These motion changes indicated a loss of protein stability, more flexibility, and less well-folded protein conformation in the case of the five mutant models (L305R, W342C, G403R, V420E, and W444C), which was consistent with the results of RMSD, Rg, SASA, RMSF, and SSE analyses. Therefore, we propose that all five mutant models significantly affected human PC function. Notably, the G403R and V420E mutants showed undesired consequences for human PC folding, thereby disrupting protein function. This hypothesis is in good concordance with the results obtained by an in-silico study from Kovács et al., which showed Ala’s transition at position 163 of human PC to a Glu (A163E) and Val (A163V) might cause abnormal folding of the EGF2 domain, which leads to impaired secretion of human PC [32].

The effect of each mutant of human PC in interaction with FVa, as a critical factor in the coagulation pathway, was assessed using HADDOCK, ClusPro, Mutabind2, and mCSM-PPI2 web servers. The data revealed that all the mutant models (L305R, W342C, G403R, V420E, and W444C) bind to FVa in a slightly deviated orientation compared to the native PC and significantly weakened the interaction between these mutants and FVa. As human PC is firmly bound to FVa to limit and inactivate FVa proteolysis, these five mutants, by disrupting the favorable essential contacts for PC functional activity, could lead to a reduction in the catalytic efficiency of human PC. A study from Nakagawa et al. discovered that the E25K mutation could reduce the binding force between the GLA domain of PC and its receptor, EPCR. As a result, the PC could not connect to the membrane surfaces, which caused impaired anticoagulation activity of the PC [19].

## Conclusion

In the current study, we conducted a comprehensive survey of the missense SNPs in the *PC* gene using computational methods. Using an in-silico approach, the most potent SNPs that could cause the disease were identified. MD simulations method was performed to deeply analyze these high-risk SNPs and determine the effect of each mutation on the human PC structure. Structural analysis of the most high-risk mutations (L305R, W342C, G403R, V420E, and W444C) after simulation revealed no significant structural changes near the mutation region. In contrast, all mutants showed higher flexibility and lower stability in the overall structure, especially in the autolysis loop. Molecular docking assessment revealed that all high-risk mutants decreased and destabilized the interaction complex of human PC with FVa, which could influence the activity of PC in the coagulation pathway. These data agree with the research of Mace et al. [92] and Marquardt et al. [93], who discovered that mutations in α-lytic and α tryptase caused no changes in the overall serine protease folding, while they showed impaired enzymatic activity. Therefore, according to this investigation, these high-risk mutants could be prospective candidates for drug design and evaluated for clinical application in patients at risk of bleeding due to impaired anticoagulant activity.

## Supporting information

S1 Fig Evolutionary conservancy of human PC produced by ConSurf tool. The higher the score, the more conserved the position. The letters “b” and “e” indicate the buried and exposed residues, respectively. the letters “f” and “s” reveal the functional and structural residues, respectively. The 23 amino acid positions corresponded to 26 high-risk missense SNPs marked by red boxes.
(TIF)

S2 Fig Multiple sequence alignment of the human full-length PC (Uniport’s ID: P04070) with its PDB structures (6M3B, 6M3C, 3F6U, 1AUT). (TIF)

S3 Fig A) The Tertiary structure of the human PC is represented in the cartoon. B) ProSA web server result of the human PC. The black dot is PC which is located in the region of protein structures that are identified as X-ray and NMR.
(TIF)

S4 Fig The RMSF plot of each mutation was aligned to the RMSF plot of the native PC, where black, blue, orange, red, green, and purple line describes native, L305R, W342C, G403R, V420E, and W444C, respectively. The colored column bars showed the different signature regions of the SP domain in human PC.
(TIF)

S1 Table Prediction of the effect of 340 missense SNPs on the human PC by various tools. (XLSX)

S2 Table Identification of high-risk missense SNPs of the human PC. (XLSX)

S3 Table The human PC model validation. (DOCX)

S4 Table Effect of 26 high-risk missense SNPs (28 mutant amino acids) on protein stability predicted by different tools. (DOCX)

S5 Table The damaging scores of the 12 most high-risk missense SNPs predicted by all methods. (XLSX)

S6 Table The damaging scores of the 12 most high-risk missense SNPs are normalized between 0 to 1 by min-max scaling. (XLSX)

S7 Table Calculated mean values for various properties based on the two replicated simulations, their standard deviations between the mean values of native PC and its mutant structures over 200 ns MD simulation times were mentioned. (DOCX)

S8 Table MD configuration system of wild type and mutant types. (DOCX)

S9 Table Percentage of residues participated in the secondary structure formation of the native form and mutants L305R, W342C, G403R, V420E, and W444C models. (DOCX)

S10 Table The effect of each mutation on protein-protein interaction was measured by HADDOCK, ClusPro, MutaBind2, and mCSM-PPI2 web servers. Furthermore, the interfacial residues of native human PC and mutant forms (L305R, W342C, G403R, V420E, and W444C) in complex with the activated form of Factor V were mentioned.
(DOCX)
