# Supplementary figures and images for: Computational analysis of the functional and structural impact of the most deleterious missense mutations in the human Protein C
Source: PLoS One. 2023 Nov 28;18(11):e0294417. doi: 10.1371/journal.pone.0294417 (PMC10683990; doi:10.1371/journal.pone.0294417)

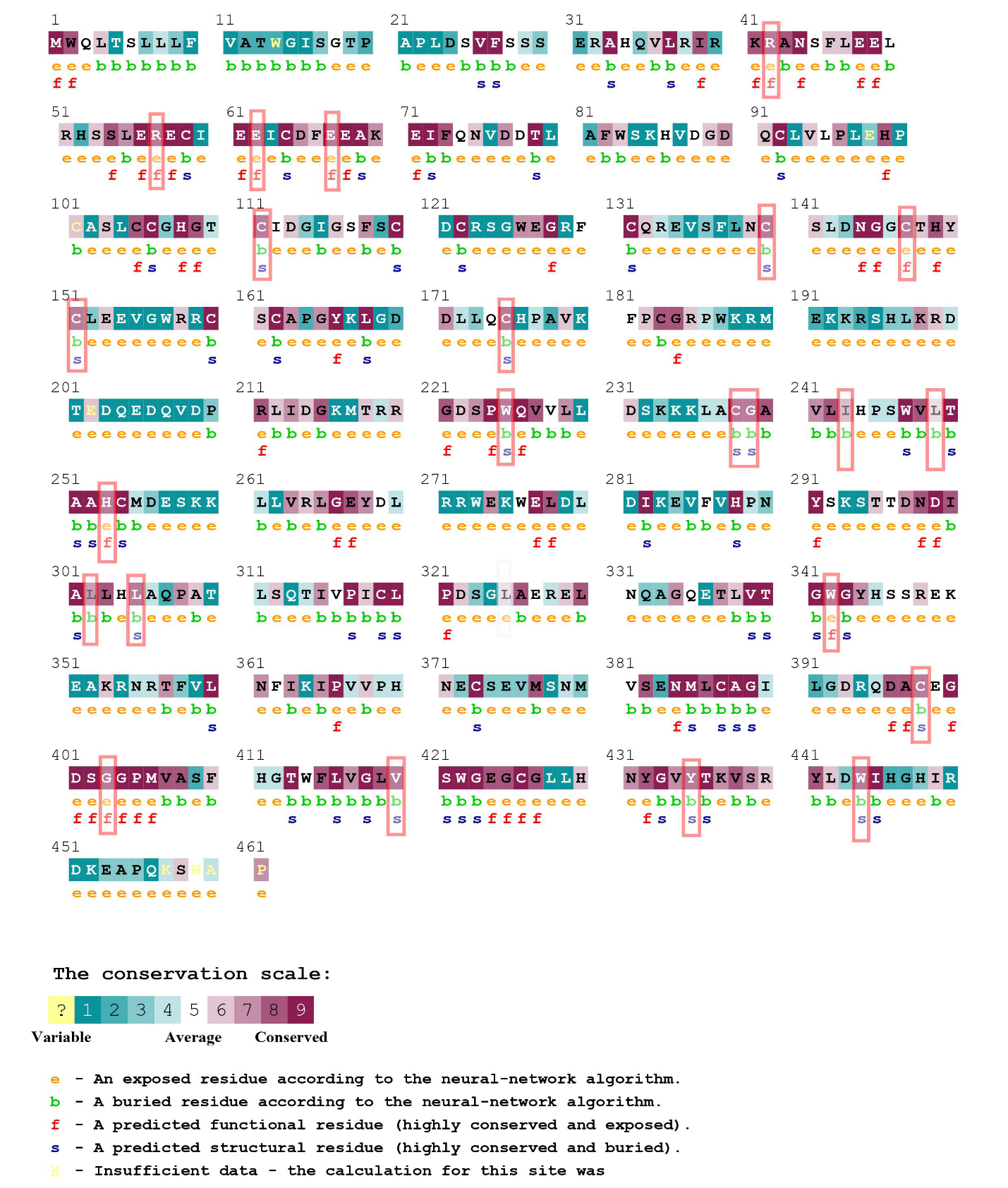

Supplement: S1 Fig — The higher the score, the more conserved the position. The letters “b” and “e” indicate the buried and exposed residues, respectively. the letters “f” and “s” reveal the functional and structural residues, respectively. The 23 amino acid positions corresponded to 26 high-risk missense SNPs marked by red boxes. (TIF) [file pone.0294417.s001.tif]

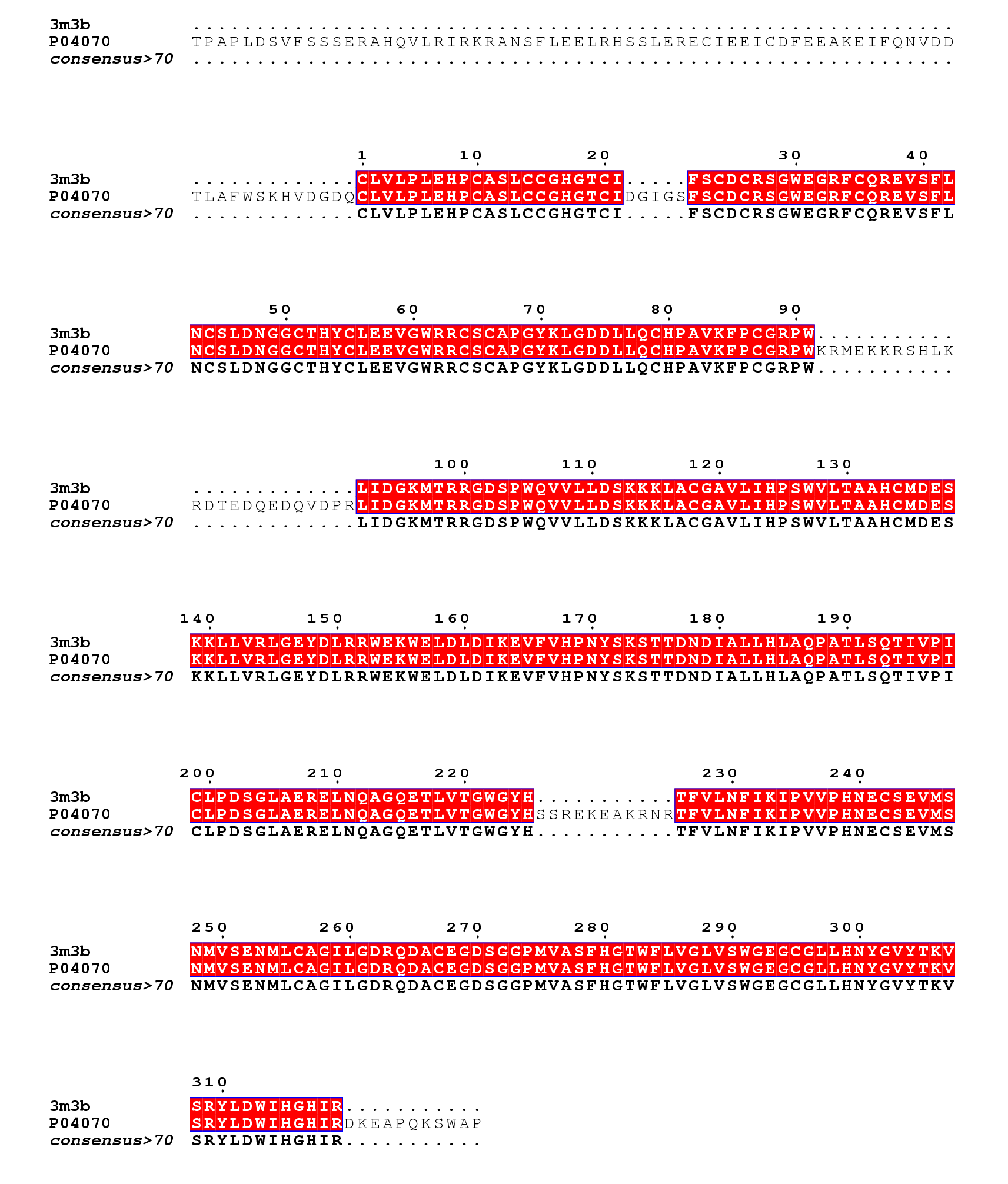

Supplement: S2 Fig — (TIF) [file pone.0294417.s002.tif]

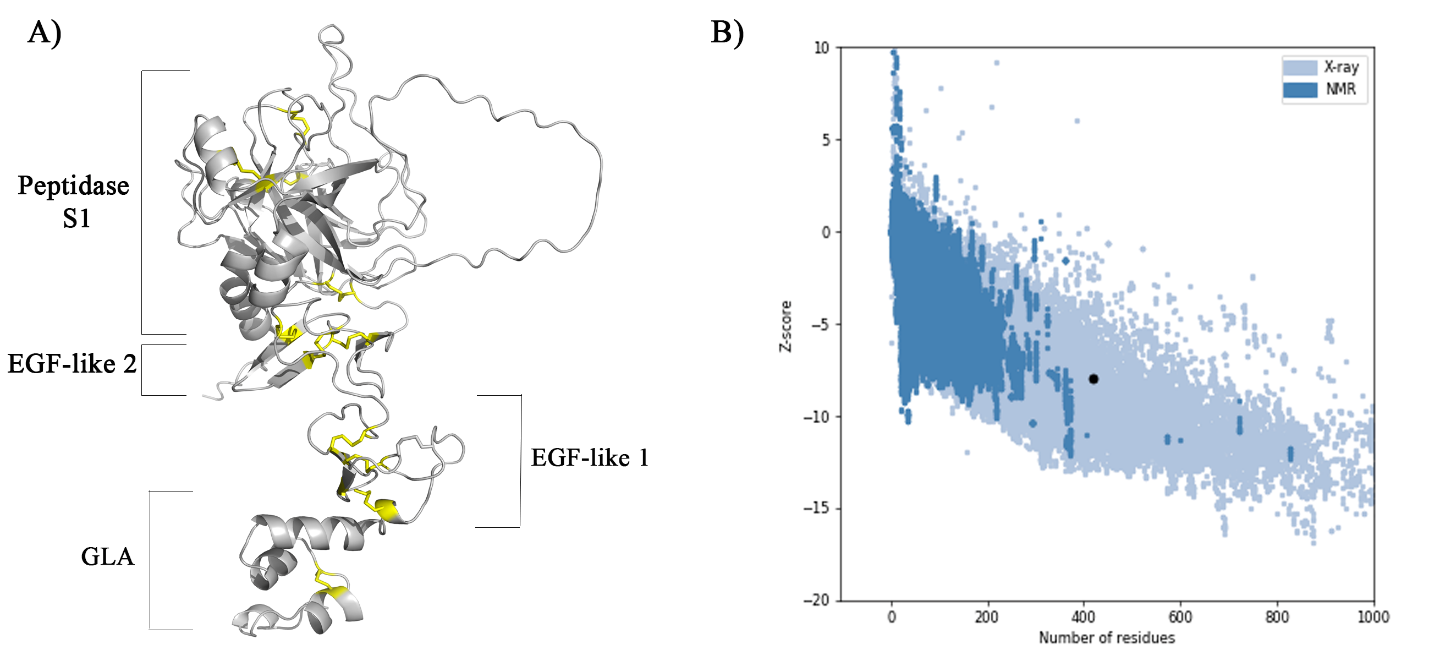

Supplement: S3 Fig — A) The Tertiary structure of the human PC is represented in the cartoon. B) ProSA web server result of the human PC. The black dot is PC which is located in the region of protein structures that are identified as X-ray and NMR. (TIF) [file pone.0294417.s003.tif]

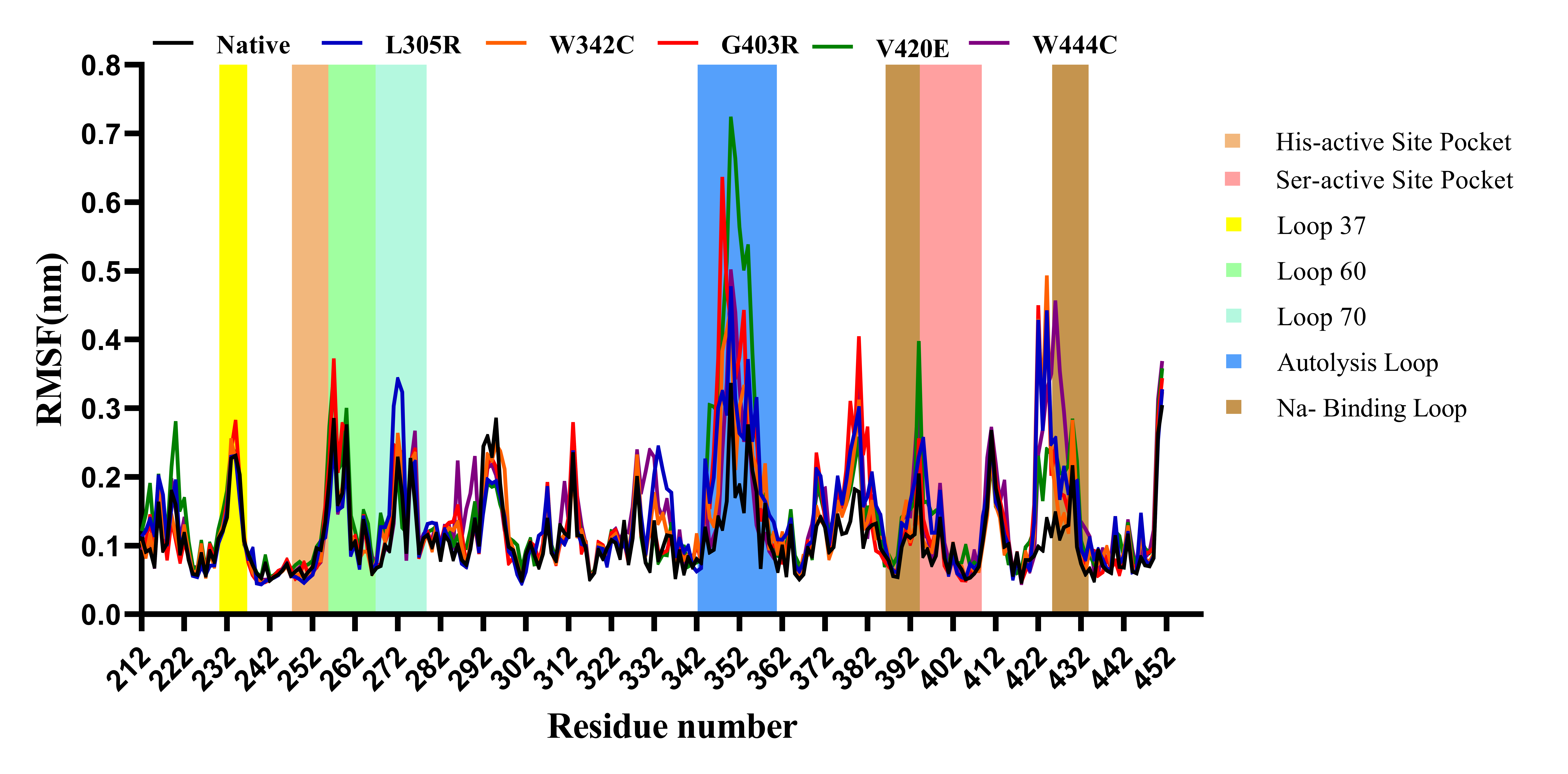

Supplement: S4 Fig — The colored column bars showed the different signature regions of the SP domain in human PC. (TIF) [file pone.0294417.s004.tif]
