# Supplementary material for: Computational analysis of the functional and structural impact of the most deleterious missense mutations in the human Protein C
Source: PLoS One. 2023 Nov 28;18(11):e0294417. doi: 10.1371/journal.pone.0294417 (PMC10683990; doi:10.1371/journal.pone.0294417)
Supplement: S3 Table — (DOCX) [file pone.0294417.s007.docx]

**S3 Table**. The human PC model validation.

| Z-score model | Procheck | | | | Varify3D | ERRAT | MolProbity |
| --- | --- | --- | --- | --- | --- | --- | --- |
|  | Core | Allow | Generally allowed | Disallowed |  |  |  |
| Native | 80.7% | 17.4% | 1.6% | 0.3% | 79.71% | 96.31 | 1.09 |
