# Supplementary material for: Computational analysis of the functional and structural impact of the most deleterious missense mutations in the human Protein C
Source: PLoS One. 2023 Nov 28;18(11):e0294417. doi: 10.1371/journal.pone.0294417 (PMC10683990; doi:10.1371/journal.pone.0294417)
Supplement: S4 Table — (DOCX) [file pone.0294417.s008.docx]

**S4 Table.** Effect of 26 high-risk missense SNPs (28 mutant amino acids) on protein stability predicted by six sequence and structure-based tools, I-Mutant 2.0, MUpro, DynaMut, CUPSAT, mCSM, and DUET. where Gibbs free energy change value (DDG) < 0 decreases stability (D), while DDG > 0 increases (I) the stability of the mutant protein. Mutations predicted as destabilizing are shown in bold.

| SNP ID | Substitution | I-Mutant 2 | MUpro | INPS-MD | DynaMut | CUPSAT | mCSM | DUET |
| --- | --- | --- | --- | --- | --- | --- | --- | --- |
| rs774572099 | **R42S** | D | D | D | D | D | D | D |
|  | **R42C** | D | D | I | D | D | D | D |
| rs757583846 | R57W | D | D | D | I | I | D | D |
| rs574949343 | R57L | D | D | I | D | I | D | D |
| rs121918148 | E62A | I | D | D | D | D | D | I |
| rs1448630830 | E67K | D | D | D | I | I | D | I |
| rs1171885932 | C111Y | D | D | D | I | D | D | D |
| rs747735192 | C140S | D | D | D | I | D | I | I |
|  | C140F | D | D | D | I | D | D | D |
| rs1247269491 | C147Y | I | D | D | D | D | D | D |
| rs767201513 | C151R | D | D | D | I | D | D | I |
| rs199469474 | C175Y | I | D | D | I | D | D | D |
| rs1277271891 | W225R | D | D | D | D | I | D | D |
| rs757925208 | **W225C** | D | D | D | D | D | D | D |
| rs748099849 | C238S | I | D | D | D | D | D | D |
| rs777486993 | **G239R** | D | D | D | D | D | D | D |
| rs749500010 | G239E | I | D | D | I | D | D | D |
| rs774584131 | **I243T** | D | D | D | D | D | D | D |
| rs375156587 | **L249P** | D | D | D | D | D | D | D |
| rs1353816203 | H253P | I | D | D | D | I | D | D |
| rs1573459781 | **L303R** | D | D | D | D | D | D | D |
| rs1241074486 | **L305R** | D | D | D | D | D | D | D |
| rs781097228 | **W342C** | D | D | D | D | D | D | D |
| rs1439742162 | C398R | D | D | D | I | D | I | I |
| rs1442363621 | **G403R** | D | D | D | D | D | D | D |
| rs760579201 | **V420E** | D | D | D | D | D | D | D |
| rs1271213613 | **Y435H** | D | D | D | D | D | D | D |
| rs121918142 | **W444C** | D | D | D | D | D | D | D |
