# Supplementary material for: Computational analysis of the functional and structural impact of the most deleterious missense mutations in the human Protein C
Source: PLoS One. 2023 Nov 28;18(11):e0294417. doi: 10.1371/journal.pone.0294417 (PMC10683990; doi:10.1371/journal.pone.0294417)
Supplement: S7 Table — (DOCX) [file pone.0294417.s011.docx]

**S7 Table**. Calculated mean values for various properties based on the two replicated simulations, their standard deviations between the mean values of native PC and its mutant structures over 200 ns MD simulation times were mentioned.

| Systems | Simulation trajectory 1 | Simulation trajectory 2 | Mean ± Standard deviation |
| --- | --- | --- | --- |
| Native  L305R  W342C  G403R  V420E  W444C | **RMSD (nm)** | | |
|  | 0.195 ± 0.015  0.221 ± 0.025  0.216 ± 0.028  0.218 ± 0.029  0.188 ± 0.021  0.199 ± 0.018 | 0.189 ± 0.028  0.208 ± 0.022  0.196 ± 0.023  0.271 ± 0.033  0.304 ± 0.057  0.241 ± 0.0161 | 0.192 ± 0.020  0.214 ± 0.023  0.206 ± 0.020  0.244 ± 0.029  0.246 ± 0.035  0.220 ± 0.014 |
| Native  L305R  W342C  G403R  V420E  W444C | **Rg (nm)** | | |
|  | 1.735 ± 0.007  1.746 ± 0.008  1.737 ± 0.006  1.742 ± 0.008  1.748 ± 0.006  1.750 ± 0.006 | 1.706 ± 0.006  1.744 ± 0.008  1.736 ± 0.008  1.788 ± 0.008  1.736 ± 0.008  1.744 ± 0.009 | 1.720 ± 0.005  1.745 ± 0.005  1.740 ± 0.006  1.765 ± 0.005  1.742 ± 0.005  1.747 ± 0.005 |
| Native  L305R  W342C  G403R  V420E  W444C | **SASA (nm^2^)** | | |
|  | 120.245 ± 2.257  126.451 ± 2.065  122.112 ± 2.11  125.577 ± 2.22  121.340 ± 2.368  125.342 ± 2.376 | 122.800 ± 1.996  125.044 ± 2.325  123.484 ± 1.968  129.185 ± 2.484  126.973 ± 2.381  124.063 ± 2.613 | 121.523 ± 1.350  125.747 ± 1.514  123.547 ± 1.472  127.381 ± 1.520  124.156 ± 1.6585  124.703 ± 1.825 |
| Native  L305R  W342C  G403R  V420E  W444C | **RMSF (nm)** | | |
|  | 0.113 ± 0.059  0.136 ± 0.077  0.144 ± 0.086  0.153 ± 0.091  0.139 ± 0.084  0.141 ± 0.069 | 0.110 ± 0.082  0.135 ± 0.076  0.124 ± 0.086  0.156 ± 0.0913  0.144 ± 0.097  0.138 ± 0.079 | 0.111 ± 0.065  0.140 ± 0.076  0.134 ± 0.086  0.154 ± 0.091  0.1421 ± 0.088  0.139 ± 0.069 |
| Native  L305R  W342C  G403R  V420E  W444C | **Covariance matrix (nm^2^)** | | |
|  | 3.779  9.419  8.772  9.387  11.017  8.052 | 7.170  9.378  6.982  11.315  11.008  7.100 | 5.475  9.399  7.877  10.351  11.013  7.576 |
