# Supplementary material for: Computational analysis of the functional and structural impact of the most deleterious missense mutations in the human Protein C
Source: PLoS One. 2023 Nov 28;18(11):e0294417. doi: 10.1371/journal.pone.0294417 (PMC10683990; doi:10.1371/journal.pone.0294417)
Supplement: S8 Table — (DOCX) [file pone.0294417.s012.docx]

**S7 Table**. MD configuration system of wild type and mutant types. The number of ions, water molecules, and atoms in each simulation system is presented.

| System | Sodium | Chlorine | Water (Molecules) | Total size (atoms) |
| --- | --- | --- | --- | --- |
|  | Sodium | Chlorine | Water (Molecules) | Total size (atoms) |
| Native | 2 | - | 5811 | 51198 |
| L305R | 1 | - | 5813 | 51208 |
| W342C | 2 | - | 5816 | 51200 |
| G403R | 1 |  | 5809 | 51208 |
| V420E | 3 | - | 5812 | 51201 |
| W444C | 2 | - | 5811 | 51185 |
