# Supplementary material for: Computational analysis of the functional and structural impact of the most deleterious missense mutations in the human Protein C
Source: PLoS One. 2023 Nov 28;18(11):e0294417. doi: 10.1371/journal.pone.0294417 (PMC10683990; doi:10.1371/journal.pone.0294417)
Supplement: S9 Table — (DOCX) [file pone.0294417.s013.docx]

**S8 Table**. Percentage of residues participated in the secondary structure formation of the native form and mutants L305R, W342C, G403R, V420E, and W444C models. Structure = A-Helix + B-Sheet + B-Bridge + Turn.

| Systems | Structure | coil | b-sheet | b-bridge | bend | turn | A-helix | 5-helix | 3-helix |
| --- | --- | --- | --- | --- | --- | --- | --- | --- | --- |
| Native | 0.57 | 0.28 | 0.31 | 0.03 | 0.12 | 0.17 | 0.06 | 0.01 | 0.03 |
| L305R | 0.54 | 0.31 | 0.28 | 0.02 | 0.13 | 0.17 | 0.07 | 0 | 0.02 |
| W342C | 0.56 | 0.29 | 0.29 | 0.03 | 0.13 | 0.17 | 0.07 | 0 | 0.02 |
| G403R | 0.55 | 0.29 | 0.29 | 0.03 | 0.12 | 0.17 | 0.06 | 0 | 0.03 |
| V420E | 0.56 | 0.29 | 0.3 | 0.03 | 0.12 | 0.16 | 0.07 | 0 | 0.03 |
| W444C | 0.54 | 0.29 | 0.3 | 0.02 | 0.12 | 0.16 | 0.06 | 0.01 | 0.03 |
