# Supplementary material for: Computational analysis of the functional and structural impact of the most deleterious missense mutations in the human Protein C
Source: PLoS One. 2023 Nov 28;18(11):e0294417. doi: 10.1371/journal.pone.0294417 (PMC10683990; doi:10.1371/journal.pone.0294417)
Supplement: S10 Table — Furthermore, the interfacial residues of native human PC and mutant forms (L305R, W342C, G403R, V420E, and W444C) in complex with the activated form of Factor V were mentioned. (DOCX) [file pone.0294417.s014.docx]

S9 Table. The effect of each mutation on protein-protein interaction was measured by HADDOCK, ClusPro, MutaBind2, and mCSM-PPI2 web servers. Furthermore, the interfacial residues of native human PC and mutant forms (L305R, W342C, G403R, V420E, and W444C) in complex with the activated form of Factor V were mentioned.

| **Complex** | **Software** | **KD** | **∆G** | **mcSM-PPI** | | **Mutabind2** | | | **RMSD** | **Fva** | **APC** |
| --- | --- | --- | --- | --- | --- | --- | --- | --- | --- | --- | --- |
|  |  |  |  | ∆G | affinity | ∆G | affinity | effect |  |  |  |
| **Native** | Haddock | 8.3e-08 | -9.7 |  |  |  |  |  | 0.5 | Lys304, Met319, Lys404, Glu323, Phe325, Leu308, Arg306, Asn307, Asp401, Arg321, Trp322, Arg505, Arg317, Ile311, Lys310 | Glu349, Leu326, Arg271, Leu270, Ser232, Lys234, Arg254, Lys235, Lys233, Glu275, Glu399, Tyr344, Thr357 |
|  | Cluspro | 1.3e-13 | -17.6 |  |  |  |  |  |  |  |  |
| **G403R** | Haddock | 3.5e-07 | -8.8 | -0.62 | decreased | 0.13 | destabilize | deleterious | 0.5 | Arg506, Glu323, Arg321, Lys404, Val406,  Trp322, Arg505, Asp373, Ser375 | Arg271, Lys235, Ser232, Asp231, Lys233, Lys234, Glu267, Glu277, Trp273, Arg272, Lys350, Glu349 |
|  | Cluspro | 3.3e-12 | -15.7 | -0.94 | decreased | 0.15 | destabilize | deleterious |  |  |  |
| **L305R** | Haddock | 4.4e-07 | -8.7 | -0.27 | decreased | 0.6 | destabilize | deleterious | 0.4 | Glu315, His318, Leu503, Arg316, Trp322, Glu323, Asp373, Glu372, Asp504, Arg505, Arg317, Arg506 | Trp273, Arg272, Ser232, Lys233, Arg271, Asp269, Asp279, Lys234, Phe358, Lys235 |
|  | Cluspro | 2.5e-12 | -15.8 | -0.29 | decreased | 0.7 | destabilize | deleterious |  |  |  |
| **V420E** | Haddock | 1.8e-07 | -9.7 | -0.34 | decreased | 0.01 | destabilize | Non-deleterious | 0.38 | Arg505, Ser375, Asp373, Met319, Arg317, Asn382, Glu387, Met385, Glu314, Glu515, Glu507, Gln370, Glu372, Thr380, Pro383, Arg506 | Thr357, Trp342, Leu236, Phe358, Trp273, Glu349, Asp279, Lys260, Ser232, Lys350, Lys233, Lys234, Arg264, Leu262, Glu267, Leu270, Tyr344, Gly343 |
|  | Cluspro | 3.1e-11 | -13.7 | -0.36 | decreased | 0.03 | destabilize | Non-deleterious |  |  |  |
| **W342C** | Haddock | 1.8e-07 | -9.7 | -0.37 | decreased | 1.06 | destabilize | deleterious | 0.56 | Arg317, Asp373, Ser375, Met319, Arg321, Arg506, Arg505, Glu323, Trp322, Lys404 | Arg281, Arg354, Lys260, Ser232, Leu270, Trp273, Lys234, Lys235, Lys233, Asp231, Glu257 |
|  | Cluspro | 5.8e-11 | -14.0 | -1.45 | decreased | 1.08 | destabilize | deleterious |  |  |  |
| **W444C** | Haddock | 6.1e-08 | -9.8 | -0.55 | decreased | 1.23 | destabilize | deleterious | 0.43 | Lys404, Trp322, Glu323, Phe325, Leu308, Asn307, Glu314, Arg317, Ile311, Met319, Arg321, Lys310, Tyr427, Arg505, Asp504 | Asn355, Leu270, Arg271, Arg272, Glu349, Glu399, Gly343, Tyr344, Lys235, Phe358, Arg354, Arg348, Ser347, Lys353, Ser232, Lys234, Lys233 |
|  | Cluspro | 6.7e-13 | -16.6 | -0.53 | decreased | 1.33 | destabilize | deleterious |  |  |  |

KD is based on Molar, ∆G is based on Kcal/mol, and RMSD is based on Angstrom.
